# Case report: Oral anticoagulant combined with percutaneous coronary intervention for peripheral embolization of left ventricular thrombus caused by myocardial infarction in a patient with diabetes mellitus

**DOI:** 10.3389/fcvm.2022.1019945

**Published:** 2022-12-08

**Authors:** Chao Zhu, Li Zhou, Hongli Gao, Jiali Wang, Jiayu Li, Hui Chen, Hongwei Li

**Affiliations:** Department of Cardiology, Beijing Friendship Hospital, Capital Medical University, Beijing, China

**Keywords:** diabetes mellitus, myocardial infarction, left ventricular thrombus, peripheral embolization, oral anticoagulant

## Abstract

**Background:**

Left ventricular thrombus (LVT) is a well-recognized complication of myocardial infarction (MI) in patients with diabetes. An embolic complication caused by LVT is a key clinical problem and is associated with worsened long-term survival.

**Case presentation:**

A 45-year-old man with persistent left abdominal pain for 1 week and left leg fatigue was admitted to the emergency department. The cause of abdominal pain was embolism of the renal artery, the splenic artery, and the superior mesenteric artery caused by cardiogenic thrombosis, which further led to splenic infarction and renal infarction. It was unclear when MI occurred because the patient had no typical critical chest pain, which may have been related to diabetic complications, such as diabetic peripheral neuropathy. Diabetes plays a pivotal role in MI and LVT formation. Because coronary angiography suggested triple vessel disease, percutaneous transluminal coronary angioplasty (PTCA) was conducted, and two drug-eluting stents were placed in the left anterior descending coronary artery (LAD). Due to a lack of randomized clinical control trials, the therapy of LVT and associated embolization has been actively debated. According to the present guidelines, this patient was treated with low-molecular-weight heparin and warfarin (oral anticoagulants) for 3 months in addition to aspirin (100 mg/day) and clopidogrel (75 mg/day) for 1 year. No serious bleeding complications were noted, and a follow-up examination showed no thrombus in the left ventricle or further peripheral thrombotic events.

**Conclusion:**

Peripheral embolization of LVT caused by MI leading to multiple organ embolization remains a rare occurrence. Diabetes plays a pivotal role in MI and LVT formation. Successful revascularization of the infarct-related coronary artery and anticoagulation therapy is important to minimize myocardial damage and prevent LVT. The present case will help clinicians recognize and manage LVT in patients with diabetes and related peripheral arterial thrombotic events with anticoagulation.

## Introduction

Left ventricular thrombus (LVT) is a well-recognized complication of acute myocardial infarction (AMI), and it is more common (15–25%) in individuals with a large, transmural anterior Q-wave infarction (1). With the widespread use of percutaneous coronary intervention (PCI) therapy over the last decades, the incidence of LVT caused by myocardial infarction (MI) has decreased. However, LVT caused by MI remains a significant cause of morbidity and mortality (2). LVT is related to an increased risk of cerebral and peripheral ischemic events and to poor long-term survival (3, 4). Multiple peripheral embolization, including the splenic artery, the renal artery, the superior mesenteric artery, and the bilateral lower extremity arteries, is an extremely rare occurrence.

Some studies indicated that diabetes mellitus, higher wall motion score index, and hypercoagulation independently increase the risk for LVT formation in patients with acute anterior MI following thrombolytic therapy (5–8). In patients with diabetes, coagulation factors are increased, while inhibitors of coagulation are decreased (9). Diabetes mellitus has been demonstrated to be a potent, independent risk factor for MI with LVT formation.

Due to a lack of randomized clinical control trials, the therapy for LVT and associated embolization has been actively debated. Successful revascularization of the infarct-related coronary artery as soon as possible is important to minimize myocardial damage and prevent LVT. Current guidelines for LVT resolution recommend anticoagulant treatment with oral anticoagulants (OACs) and vitamin K antagonists (VKAs) for 3 months (10) or 6 months (11). Here, we present a rare case of splenic infarction and bilateral renal infarction resulting from multiple peripheral embolization of LVT in a patient with diabetes mellitus who was treated successfully with OAC and intervention therapy.

## Case description

A 45-year-old man with persistent left abdominal pain for 1 week was admitted to the emergency department. He denied any chronic illness history and regular physical check-ups. For 3 days prior to admission, the patient felt fatigue and had numbness in his left leg below the ankle. The smoking history of the patient was more than 20 years with three to four cigarettes per day, and his history of drinking was more than 10 years with 1,000 ml of beer per day. His weight was 80 kg (BMI 24.07 kg/m^2^), and his abdominal girth was 106 cm. The blood pressure of the patient on admission was 150/108 mmHg, with a pulse rate of 91 beats per minute and a pulse oxygen level of 99% (indoor air). Physical assessment revealed pressing pain in the middle of the left abdomen with no rebound pain as well as muscle tension, percussion pain in the left kidney area, and weak fluctuation of the bipedal dorsal artery pulse.

Laboratory tests provided the following results: a white blood cell (WBC) count of 7.68 × 10^9^/L (normal range 3.5–9.5 × 10^9^/L); a platelet (PLT) count of 397 × 10^9^/L (normal range 125–350 × 10^9^/L); hemochrome (HGB) of 154 g/L; and a weak positive fecal occult blood (OB) test, which turned negative after subsequent reexamination. The myocardial enzyme markers (creatine kinase isoenzyme index and troponin I or T) and NT-proBNP levels were within the normal range. Renal function showed mild impairment, with a creatinine (Cr) level of 105.0 μmol/L (normal range 41–111 μmol/L) and an estimated glomerular filtration rate (eGFR) of 60.54 ml/min/1.73 m^2^. The D-dimer level was slightly elevated with a value of 1.80 mg/L (normal range 0–1.5 mg/L). The patient also had high blood sugar (Glu) and elevated blood lipids. The serum lipid profile of the patient was as follows: total cholesterol level (CHOL) of 8.13 mmol/L (normal range 3.9–5.2 mmol/L); triglyceride (TG) level of 2.10 mmol/L (normal range 0.57–1.7 mmol/L); and low-density lipoprotein cholesterol (LDL-c) level of 4.97 mmol/L (normal range 2.34–3.12 mmol/L). At admission, the blood sugar and glycosylated hemoglobin (HbA1c) levels of the patient were 23.33 mmol/L (normal range 3.92–6.16 mmol/L) and 11.70% (normal range 4.27–6.07), respectively, and an oral glucose tolerance test (OGTT) confirmed diabetes mellitus. Fundus examination (Supplementary Figures 1A,B) showed vitreous hemorrhage on the right eye, macular edema on the left eye, and skin pigmentation on both lower limbs (Supplementary Figure 1C), which indicated long-term diabetic lesions with retinopathy and dermopathy. Laboratory tests for hypercoagulability (proteins C and S) were performed with normal results.

**Figure 1:**
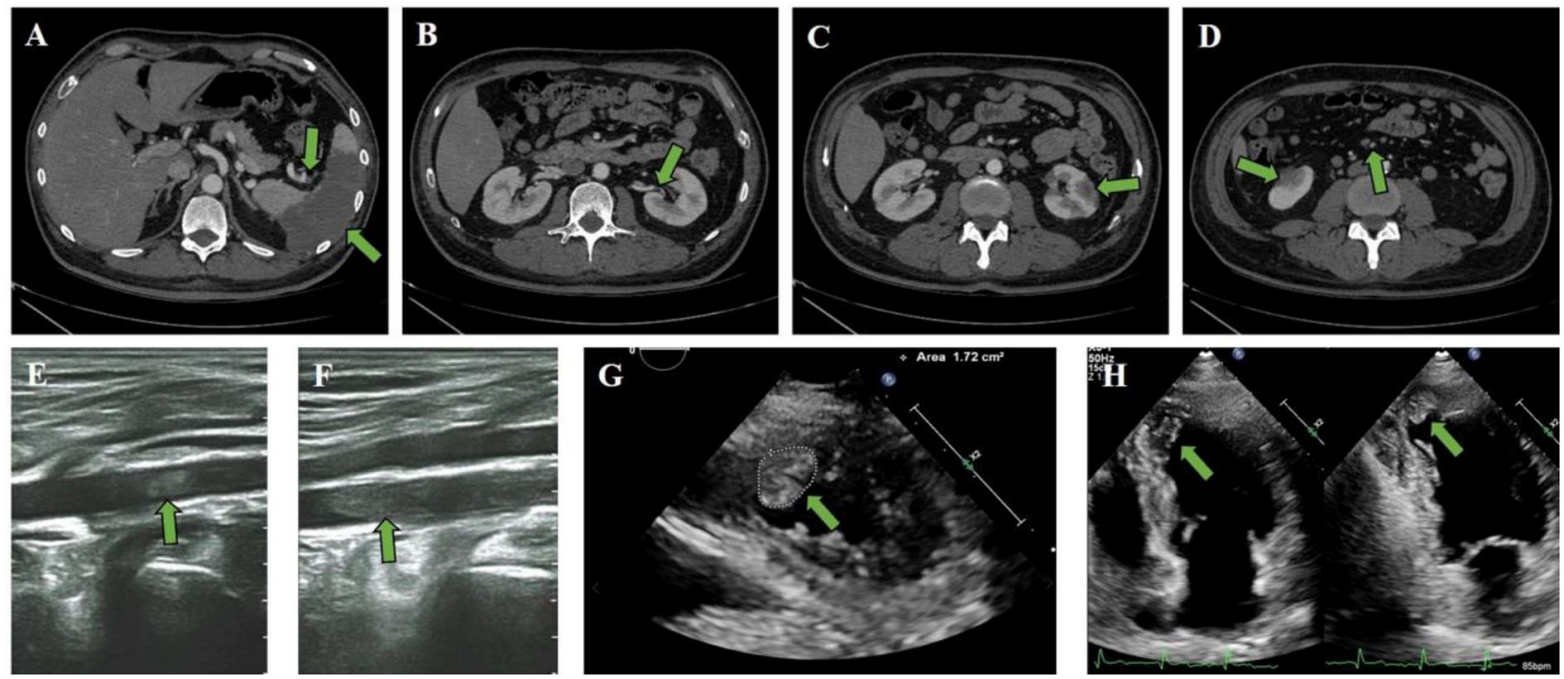
Clinical CT scan and ultrasound imaging. **(A–F)** Enhanced CT scan revealed **(A)** splenic infarction and splenic artery thrombosis, as well as **(B)** left renal artery embolization, **(C)** left renal infarction, and **(D)** superior mesenteric artery thrombosis and right renal infarction. Ultrasonography of the lower limb artery revealed **(E)** right popliteal artery thrombosis and **(F)** left posterior tibial artery thrombosis. **(G,H)** The attached thrombus (size 1.13 × 1.77 cm and area 1.72 cm^2^) was observed in the apex part of the LV.

Enhanced computed tomography (CT) scan of the abdomen and the pelvis of the patient revealed splenic infarction and splenic artery thrombosis (Figure 1A), as well as left renal artery embolization (Figure 1B), bilateral renal infarction (Figures 1C,D), and superior mesenteric artery thrombosis (Figure 1D). Ultrasonography of the lower limb artery revealed the bilateral popliteal artery and left posterior tibial artery thrombosis (Figures 1E,F). In a patient with multiple peripheral thromboembolic events, cardiogenic thrombosis should be taken into consideration. Furthermore, echocardiography showed abnormal ventricular wall motion with LVT (size of 1.13 × 1.77 cm and area of 1.72 cm^2^) at the apex to the inferior part (Figures 1G,H; Supplementary Video 1). Wall motion was observed in the middle segment of the ventricular septum and anterior wall. Hypokinesis to akinesis in LV motion was observed lateral of the basal to apical segments of the LV inferior wall, with a left ventricular ejection fraction (LVEF) decrease of 48%.

**Figure 2:**
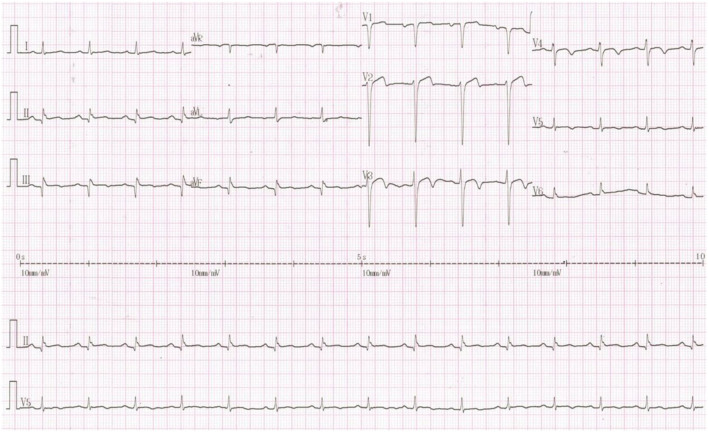
Twelve-lead ECG findings on admission. The following ECG findings were observed: sinus rhythm; Q waves in the II, III, and aVF leads; the Q waves of the inspiratory phase became shallow but still existed; poor R-wave progression in chest leads; and T-wave inversion in leads V2 to V5.

The following electrocardiogram (ECG) findings were observed: sinus rhythm; Q waves in the II, III, and augmented vector foot (aVF) leads; the Q waves of the inspiratory phase became shallow but still existed; poor R-wave progression in chest leads; and T-wave inversion in leads V2 to V5 (Figure 2).

**Figure 3:**
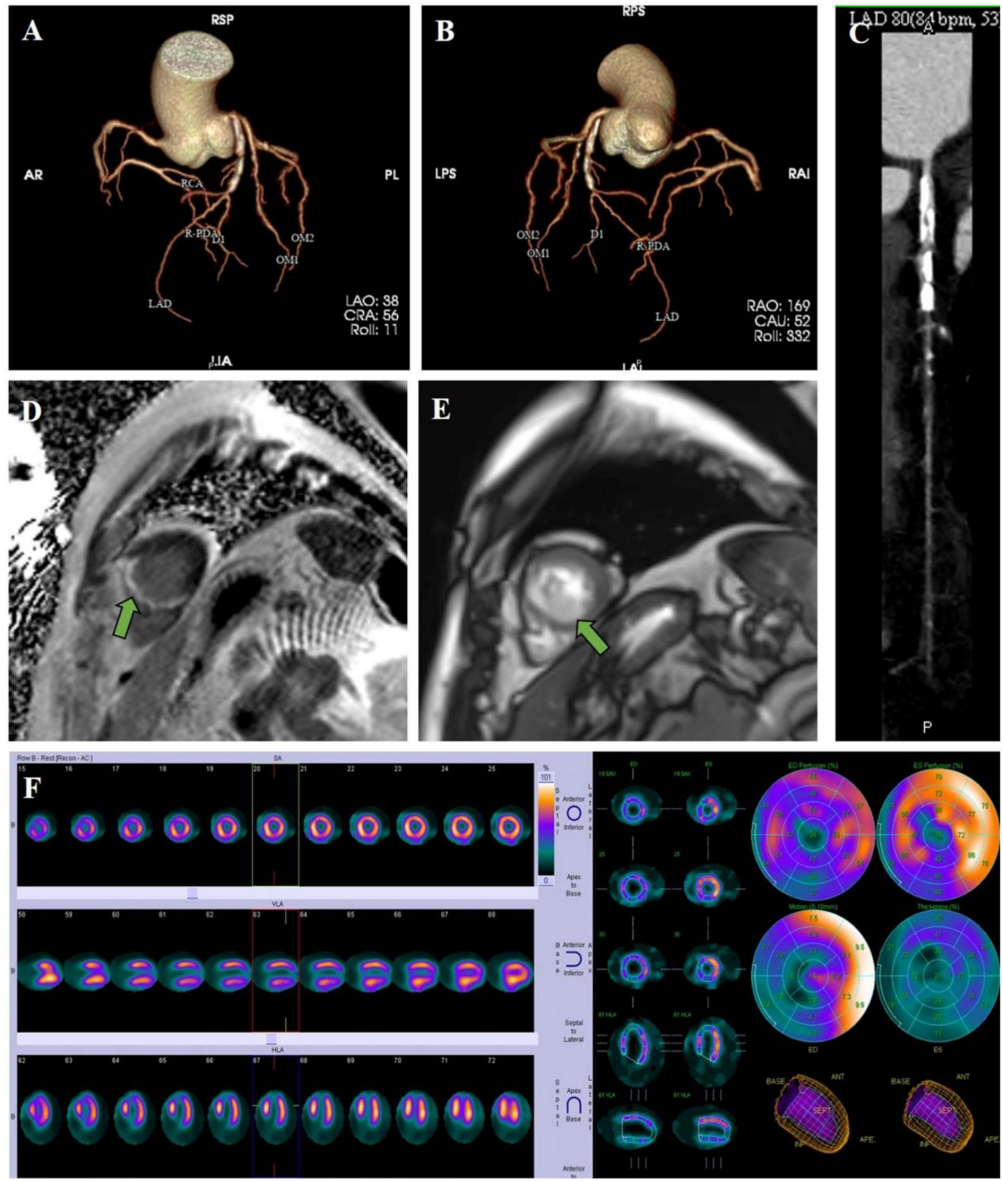
Clinical imaging. **(A–C)** A coronary CT scan showed that the proximal anterior descending artery had severe calcification and stenosis. **(D)** Cardiac magnetic resonance imaging revealed delayed enhancement on the apical septal and inferior walls, which presented transmural MI. **(E)** Cardiac magnetic resonance imaging confirmed the presence of a thrombus at the apex. **(F)** Resting myocardial radionuclides showed transmural MI and blood perfusion decrement.

The patient was treated with low-molecular-weight heparin and warfarin, with a target international normalized ratio (INR) of 1.8–2.5. Invasive procedures could not be performed due to the presence of LVT. To evaluate coronary artery and myocardial necrosis, a coronary CT scan, myocardial magnetic resonance, and radionuclide imaging were performed. The coronary CT scan showed that the proximal anterior descending artery had severe calcification and stenosis (Figures 3A–C). Subsequent cardiac magnetic resonance imaging confirmed the presence of an apical aneurysm with mobile thrombus at the apex and transmural MI on the apical septal and inferior walls (Figures 3D,E). Resting myocardial radionuclides showed transmural MI in the apical segment of the left ventricle and apical septal segments [approximately 7% of the left ventricular (LV) wall], and blood perfusion was decreased in the inferior wall of the left ventricle (Figure 3F).

**Figure 4:**
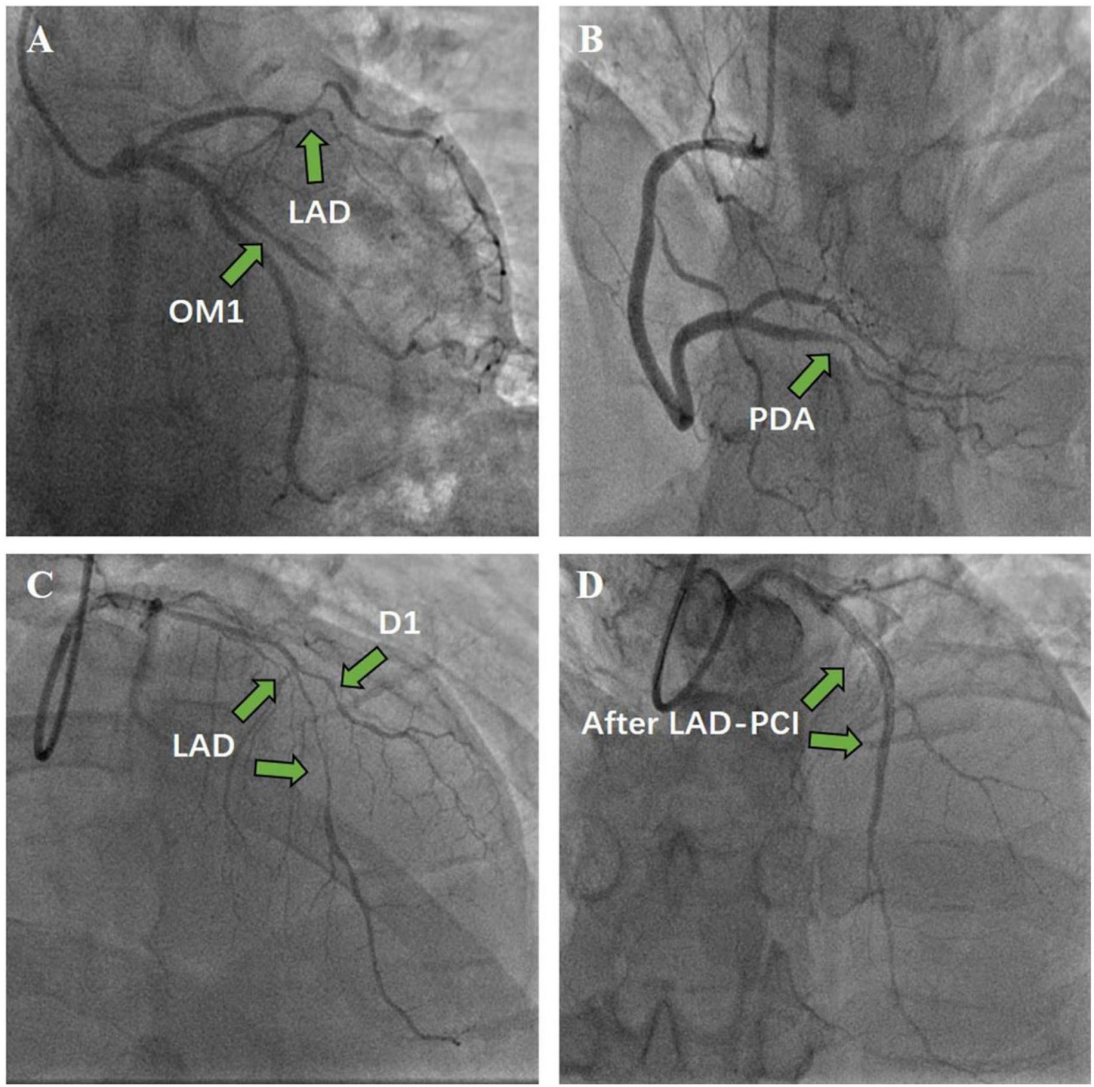
Coronary angiography and percutaneous coronary intervention. **(A)** Left coronary angiography. **(B)** Right coronary angiography. **(C)** Before stent. **(D)** Two drug-eluting stents were implanted in the LAD to achieve revascularization.

The patient was also treated with aspirin (100 mg/day), clopidogrel (75 mg/day), and proton-pump inhibitors (PPIs) to prevent gastrointestinal bleeding events. After 2 weeks of antithrombotic treatment with warfarin, aspirin, and clopidogrel, follow-up echocardiography revealed that the thrombus had almost disappeared. Because the coronary angiography suggested triple vessel disease, percutaneous transluminal coronary angioplasty (PTCA) was conducted, and two drug-eluting stents were placed in the left anterior descending coronary artery (LAD) (Figure 4).

After PCI, antithrombotic therapy with warfarin, aspirin, and clopidogrel was essential for the patient. A repeat echocardiogram revealed that the LV apical thrombus disappeared, and an enhanced CT scan showed peripheral embolism of multiple organs with no further aggravation compared to the previous scan. Diabetes was controlled with a combination of diet and insulin, acarbose, and dapagliflozin. The patient was discharged after a normal physical exam and was prescribed warfarin (3 mg/day), aspirin (100 mg/day), and clopidogrel (75 mg/day) for anticoagulation. With regular outpatient follow-ups, echocardiography showed no thrombus in the LV. Moreover, the patient was prescribed warfarin for 3 months and dual antiplatelet therapy (DAPT) for 1 year.

## Discussion

Left ventricular thrombus is a well-recognized complication of AMI. Approximately 15% of LVT cases are caused by MI, and the occurrence rate of LVT in anterior MI is even higher (1). Adverse consequences of LVT include embolic events, especially stroke, and multiple embolization of peripheral arteries. Embolic events cause poor clinical prognoses and affect the quality of life of patients.

The following pathophysiology leading to LVT can be explained by Virchow's triad: hypercoagulable state, ventricular aneurysm stasis, ventricular endothelial injury, and ejection fraction decrease after MI (12). LVT increases the risk of major cardiovascular events and mortality, leading to poorer long-term survival. Short-term thrombus dissolution by anticoagulants can improve the prognosis of patients.

The underlying endomyocardial pathological changes and low regional intracardiac blood flow velocity caused by MI are the most important mechanisms of thrombus formation (13, 14). In most instances, a hypercoagulable state may also contribute to some extent (5, 6). In diabetes patients with increased plasma lipid levels, coagulation factors such as fibrinogen, factor VII, factor VIII, von Willebrand's factor, and factor X are increased, but inhibitors of coagulation such as antithrombin II, protein C, and protein S are decreased (9, 15). Diabetes plays a pivotal role in MI and LVT formation.

In the present case, the patient presented with abdominal pain as the first symptom, which was attributed to embolism of the renal artery, the splenic artery, and the superior mesenteric artery caused by cardiogenic thrombosis, further leading to splenic infarction and renal infarction. It was unclear when the MI occurred because the patient had no typical critical chest pain, which may have been due to diabetic complications, such as diabetic peripheral neuropathy. Changes in retinopathy and dermopathy indirectly indicate a long history of diabetes. Diabetic peripheral neuropathy is one of the most common long-term complications of diabetes and is associated with cardiovascular risk factors and mortality. The ECG and magnetic resonance imaging of the patient suggested an old anterior MI. The LVT can form within several days after the MI, and the hypokinesis to akinesis in LV motion throughout the entire myocardium may have enhanced thrombus formation (15).

Due to a lack of randomized clinical control trials, the management of LVT and associated embolization has been actively debated (12, 16). Anticoagulation therapy has been shown to reduce the risk of embolic complications in patients with LVT, but no prospective randomized study has been performed (17). Current guidelines for LVT resolution recommend anticoagulant treatment with VKAs for 3 or 6 months (8, 18). Larger infarct sizes have greater risks of forming into thrombosis. A larger infarction can lead to myocardial injury, inflammatory response, hypercoagulable state, and abnormal wall motion (7, 19). The incidence of LVT early after AMI is low if primary PCI with stenting is successfully performed to salvage the myocardium from dying. Thus, successful revascularization of the “criminal” coronary artery is important to minimize the MI area and prevent LVT. Importantly, dual antiplatelet treatment after PCI has no or only limited influence on thrombin generation, which plays a key role in the development of LVT (20).

Triple antithrombotic therapy increases the risk of bleeding, especially considering the patient's history of hypertension and positive fecal occult blood (OB) test in our case. Therefore, the anticoagulant therapy strategy and duration are important. However, the optimal duration of triple antithrombotic therapy is controversial, especially after PCI and DAPT. The current consensus suggests that patients with LVT should be prescribed OAC with VKA therapy for up to 6 months (18). However, this recommendation does not consider the need for DAPT after stenting. VKAs and DAPT may increase the risk of bleeding, and the optimal duration of triple antithrombotic therapy is decided by bleeding risk and stent thrombus. If echocardiography or cardiac magnetic resonance imaging shows no thrombi after 3 months of antithrombotic therapy, OAC can be stopped earlier than 6 months, especially if the abnormal wall motion is improved (21). Some authors recommended that patients after PCI who have LVT or are at risk of LVT with apical akinesis or dyskinesis should be prescribed VKAs for up to 3 months and that the DAPT duration should be based on the type of stent (22). According to the present guidelines, the present case of LVT and peripheral embolization was treated with low-molecular-weight heparin and warfarin (OAC) in addition to aspirin and clopidogrel. The patient was rated as having a high risk for bleeding according to the CRUSADE score for bleeding risk. With a high bleeding risk and improved wall motion, the patient was treated with warfarin (OAC) for 3 months and DAPT for 1 year after PCI. The patient is currently only taking one antiplatelet drug (clopidogrel). The patient did not experience any serious bleeding complications after treatment with warfarin and DAPT. The anticoagulant therapy strategy and duration were important based on the risk of ischemia and bleeding. Here, we present successful anticoagulation therapy for LVT in a patient with diabetes and related peripheral arterial thrombotic event, providing a reference for similar cases.

## Conclusion

Left ventricular thrombus formation is a serious complication of MI, and peripheral embolic events caused by LVT are related to poor long-term survival. Multiple embolization due to LVT leading to splenic infarction, bilateral renal infarction, and bilateral popliteal artery thrombus is rare. The presence of wall motion abnormalities, MI location, and MI size is the most powerful independent predictors of LVT formation. In the present case, the patient suffered from an asymptomatic MI, and the infarct-related coronary artery was successfully revascularized through PCI. Diabetes plays a pivotal role in asymptomatic MI and LVT formation. The present patient was treated with low-molecular-weight heparin followed by warfarin for 3 months in addition to aspirin and clopidogrel for 1 year. The anticoagulation treatment prevented further thromboembolic events and caused the LVT to disappear without any serious bleeding complications. The present case will help clinicians recognize and manage LVT in patients with diabetes and related peripheral arterial thrombotic events with anticoagulation. More studies on anticoagulation therapy in patients with LVT are needed to improve long-term quality of life and reduce morbidity and mortality.

## Data availability statement

The original contributions presented in the study are included in the article/Supplementary material, further inquiries can be directed to the corresponding authors.

## Ethics statement

The studies involving human participants were reviewed and approved by Ethics Committee Board of the Beijing Friendship Hospital, Capital Medical University. The patients/participants provided their written informed consent to participate in this study. Written informed consent was obtained from the individual(s) for the publication of any potentially identifiable images or data included in this article.

## Author contributions

CZ, LZ, and HG performed patient management and data collection. CZ, JW, and JL drafted the manuscript and edited the figures. LZ and HC performed the angioplasty. HC and HL critically revised the manuscript for important intellectual content. All authors contributed to the article and approved the submitted manuscript.

## Funding

The research was supported by the Youth Program of the National Natural Science Foundation of China (Grant No. 82000311) and the National Key R&D Program of China (Grant No. 2021ZD0111004).

## Conflict of interest

The authors declare that the research was conducted in the absence of any commercial or financial relationships that could be construed as a potential conflict of interest.

## Publisher's note

All claims expressed in this article are solely those of the authors and do not necessarily represent those of their affiliated organizations, or those of the publisher, the editors and the reviewers. Any product that may be evaluated in this article, or claim that may be made by its manufacturer, is not guaranteed or endorsed by the publisher.
