# Supplementary material for: Case report: Oral anticoagulant combined with percutaneous coronary intervention for peripheral embolization of left ventricular thrombus caused by myocardial infarction in a patient with diabetes mellitus
Source: Front Cardiovasc Med. 2022 Dec 8;9:1019945. doi: 10.3389/fcvm.2022.1019945 (PMC9775277; doi:10.3389/fcvm.2022.1019945)
Supplement: Supplementary file 1 [file Data_Sheet_1.docx]

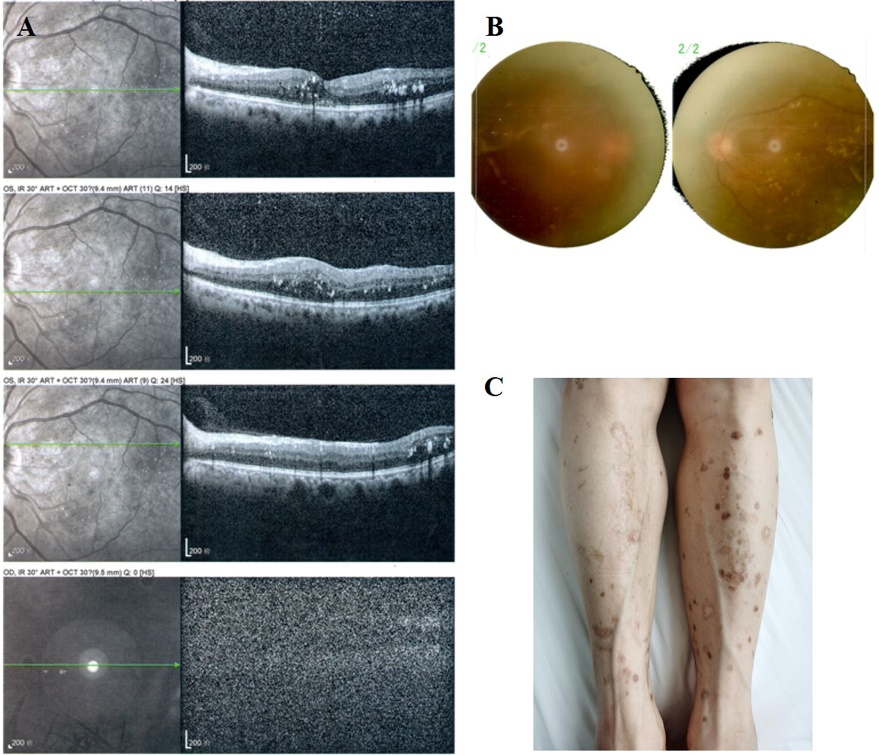


Additional files: FIGURE1 Diabetic lesion with retinopathy and dermopathy. (A) Fundus optical coherence tomography (OCT). (B) Eye-ground photography examination. (C)Lower limbs skin pigmentation.
